# Identification of QTLs for Domestication-Related Traits in Zombi Pea [*Vigna vexillata* (L.) A. Rich], a Lost Crop of Africa

**DOI:** 10.3389/fgene.2020.00803

**Published:** 2020-09-18

**Authors:** Kitiya Amkul, Prakit Somta, Kularb Laosatit, Lixia Wang

**Affiliations:** ^1^Department of Agronomy, Faculty of Agriculture at Kamphaeng Saen, Kasetsart University, Kamphaeng Saen Campus, Nakhon Pathom, Thailand; ^2^Center of Excellence on Agricultural Biotechnology: (AG-BIO/PERDO-CHE), Bangkok, Thailand; ^3^Institute of Crop Sciences, Chinese Academy of Agricultural Sciences, Beijing, China

**Keywords:** zombi pea, *Vigna vexillata*, domestication syndrome, QTL, legume

## Abstract

Zombi pea [*Vigna vexillata* (L.) A. Rich] is a legume crop found in Africa. Wild zombi pea is widely distributed throughout the tropical and subtropical regions, whereas domesticated zombi pea is rarely cultivated. Plant domestication is an evolutionary process in which the phenotypes of wild species, including seed dormancy, pod shattering, organ size, and architectural and phenological characteristics, undergo changes. The molecular mechanism underlying the domestication of zombi pea is relatively unknown. In this study, the genetic basis of the following 13 domestication-related traits was investigated in an F_2_ population comprising 198 individuals derived from a cross between cultivated (var. *macrosperma*) and wild (var. *vexillata*) zombi pea accessions: seed dormancy, pod shattering, days-to-flowering, days-to-maturity, stem thickness, stem length, number of branches, leaf area, pod length, 100-seed weight, seed width, seed length, and seeds per pod. A genetic map containing 6,529 single nucleotide polymorphisms constructed for the F_2_ population was used to identify quantitative trait loci (QTLs) for these traits. A total of 62 QTLs were identified for the 13 traits, with 1–11 QTLs per trait. The major QTLs for days-to-flowering, stem length, number of branches, pod length, 100-seed weight, seed length, and seeds per pod were clustered in linkage group 5. In contrast, the major QTLs for seed dormancy and pod shattering belonged to linkage groups 3 and 11, respectively. A comparative genomic analysis with the cowpea [*Vigna unguiculata* (L.) Walp.] genome used as the reference sequence (i.e., the genome of the legume species most closely related to zombi pea) enabled the identification of candidate genes for the major QTLs. Thus, we revealed the genomic regions associated with domestication-related traits and the candidate genes controlling these traits in zombi pea. The data presented herein may be useful for breeding new varieties of zombi pea and other *Vigna* species.

## Introduction

Plant domestication is a process in which wild forms of crop (cultivated) plants evolve and adapt to agricultural conditions and human needs via conscious and unconscious selections by humans and natural selection due to environmental factors. Domestication is one of the most important technological innovations in human history (Doebley et al., 2006). Although the processes and spatiotemporal requirements for domestication vary among crops, the processes generally lead to similar results regarding morphological and phenological changes to the wild forms. The changes from wild species to domesticated/cultivated species are collectively referred to as the “domestication syndrome” (Hammer, 1984). Compared with wild forms, domesticated forms exhibit a lack of or decreased seed dormancy and seed dispersal, produce larger organs (fruit, seed, leaf, and stem), flower and mature earlier, and have a more robust and firm plant architecture. During the last 20 years, plant domestication studies have become a major part of crop evolution and adaptation research. In fact, domestication is the earliest form of plant breeding (Doebley et al., 2006). The information generated in investigations of the evolutionary relationships between crops and their wild progenitors may be useful for exploiting wild plants for crop improvement (Vaughan et al., 2007). The genetic basis of the domestication syndrome of several plant species, but mainly cereals and legumes, including rice, maize, wheat, sorghum, soybean, common bean, and cowpea, has been investigated via molecular genetic analyses, especially quantitative trait locus (QTL) mapping. Several genes controlling the domestication-related traits of some of these crops have been identified and cloned (Doebley et al., 2006; Vaughan et al., 2007; Ishimaru et al., 2013).

Zombi pea [*Vigna vexillata* (L.) A. Rich] is an underutilized legume crop. Wild zombi pea is widely distributed in Africa, Asia, Australia, and the Americas (Dachapak et al., 2017). Because of its wide distribution, several wild forms of zombi pea have been identified and described (Maxted et al., 2004). Additionally, there are two types of cultivated zombi pea (i.e., seed and tuberous root) (Ferguson, 1954; Karuniawan et al., 2006). The cultivated seed type is grown in Africa (Ferguson, 1954), whereas the cultivated tuberous root type exists only in Bali, Indonesia, and East Timor (Karuniawan et al., 2006). The former is grown principally for its seeds, although the tuberous roots are sometimes consumed, whereas the latter is cultivated mainly for its tuberous roots. An analysis of molecular diversity based on microsatellite (i.e., simple sequence repeat) markers revealed that these two forms were domesticated independently (Dachapak et al., 2017). There exists a strong crossing barrier between the cultivated tuberous root type and the cultivated seed type/wild forms of zombi pea.

The genus *Vigna* includes as many as nine cultivated/domesticated species and several wild species that are used as human food, animal feed, and cover crops. Several comparative genomic analyses revealed that the genomes of *Vigna* species are highly conserved (Chaitieng et al., 2006; Isemura et al., 2007, 2010; Dachapak et al., 2018; Amkul et al., 2019; Yundaeng et al., 2019). We previously developed linkage maps of zombi pea and determined that its genome is highly conserved with those of cowpea [*Vigna unguiculata* (L.) Walp.], mung bean [*Vigna radiata* (L.) Wilczek], azuki bean [*Vigna angularis* (Willd.) Ohwi and Ohashi], and rice bean [*Vigna umbellata* (Thunb.) Ohwi and Ohashi] (Dachapak et al., 2018, Dachapak et al., 2019; Amkul et al., 2019). A recent study proved that the zombi pea genome and the genomes of common bean (*Phaseolus vulgaris* L.) and soybean [*Glycine max* (L.) Merr.] (Amkul et al., 2019) are also highly conserved. Additionally, QTLs controlling domestication-related traits have been identified in *Vigna* species, including mung bean (Isemura et al., 2012), azuki bean (Isemura et al., 2007), rice bean (Isemura et al., 2010), cowpea (Kongjaimun et al., 2012; Lo et al., 2018), and zombi pea (Dachapak et al., 2018). Although these studies indicated that the QTLs for the same traits are generally conserved among *Vigna* species, the major QTLs for some important traits, such as seed size and weight, vary between species.

The genetic basis of zombi pea domestication is particularly interesting because the two cultivated types were likely domesticated from the same or different wild zombi pea species via different processes (Dachapak et al., 2017). The seed-type zombi pea plant produces relatively few branches and exhibits a determinate growth habit. In contrast, the tuberous root-type zombi pea is a highly branched, vine-like plant with an indeterminate growth habit. Dachapak et al. (2018) identified QTLs for the domestication of the tuberous root form of zombi pea. However, because of the sterility of the mapping population, the QTLs of key domestication-related traits, including pod dehiscence (pod shattering) and seed dormancy, were not detected (Dachapak et al., 2018). Therefore, identifying the QTLs controlling domestication-related traits in the seed-type zombi pea may provide important insights regarding the genetic basis of the domestication of this species and other *Vigna* species, which may facilitate the identification of new candidate genes for crop improvement. The objectives of this study were to (i) detect the QTLs associated with domestication-related traits and (ii) identify candidate genes for these traits in the seed-type zombi pea.

## Materials and Methods

### Mapping Population

An F_2_ population comprising 198 individuals developed from a cross between TVNu 240 and TVNu 1623 was used in this study. Specifically, TVNu 240 is a cultivated zombi pea (*V. vexillata* var. *macrosperma*) accession from the Central African Republic, whereas TVNu 1623 is a wild zombi pea (*V. vexillata* var. *vexillata*) accession from Nigeria. The F_2_ population was previously used for developing high-density linkage maps and for the mapping of QTLs for bruchid (*Callosobruchus* spp.) resistance (Amkul et al., 2019). Briefly, the F_2_ plants and 10 plants of each parent were grown under field conditions, with 0.75 m × 0.75 m spacing, at Kasetsart University, Kamphaeng Sean Campus, Nakhon Pathom, Thailand between December 2017 and March 2018.

### Measurement of Domestication-Related Traits

Thirteen domestication-related traits were assessed in the F_2_ and parental plants (Table 1). All of the traits, except for leaf area (LFA), were analyzed in previous studies on *Vigna* species, including mung bean (Isemura et al., 2012), azuki bean (Isemura et al., 2007), rice bean (Isemura et al., 2010), cowpea (Kongjaimun et al., 2012), and zombi pea (Dachapak et al., 2018). The LFA of nine fully expanded leaves was measured with the LI-3100C area meter (LI-COR Biosciences, United States) at 50 days after sowing. Stem length (STL) and stem thickness (STT) were recorded at harvest. The number of days from sowing to first flowering (FLD) and the number of days from sowing to harvesting of the first pod (PDDM) were also recorded. The mature pods and seeds of each plant were harvested separately to analyze the pod-related and seed-related traits. Specifically, pod length (PDL) was measured and the number of seeds per pod (SDNPPD) was determined based on 10 pods. Pod shattering was evaluated by counting the number of twists along the pod (PDT) of five pods maintained at room temperature. The seed length and width (SDL and SDW, respectively) were recorded as the average value of five seeds. The 100-seed weight (SD100WT) was measured twice using intact seeds. Seed dormancy was assessed by measuring seed water absorption (SDWA). Specifically, 40 intact seeds harvested from each plant were immersed in water in a plastic container and incubated at 25°C for 7 days. The number of seeds that imbibed water was recorded.

**TABLE 1:** Domestication-related traits in the F_2_ population derived from a cross between TVNu 240 (cultivated) and TVNu 1623 (wild).

**General attribute**	**Organ**	**Trait**	**Trait abbreviation**	**QTL**	**Evaluation**
Pod dehiscence	Pod	Number of twists (count)	PDT	*Pdt*	Number of twists along the length of shattered pod
Seed dormancy	Seed	Water absorption (%)	SDWA	*Sdwa*	Seeds that absorbed water at 7 days after sowing at room temperature
Gigantism	Seed	100-seed weight (g)	SD100WT	*Sd100wt*	Weight of 100 seeds
		Length (mm)	SDL	*Sdl*	Maximum distance from top to bottom of the seed
		Width (mm)	SDW	*Sdw*	Maximum distance from hilum to its opposite side
	Pod	Length (mm)	PDL	*Pdl*	Length of straight pod
	Stem	Thickness (mm)	STT	*Stt*	Stem diameter under the primary leaf (measured at flowering stage)
	Leaf	Leaf area	LFA	*Lfa*	Distance from pulvinus to leaf tip
Plant type	Stem	Length (up to 10th node) (cm)	STL	*Stl*	Length from node on primary leaf to node 10 of trifoliate leaf
	Branch	Number of branches	BRNPP	*BRNPP*	Number of branches on the main stem
Earliness	Flower	Days to first flower (day)	FLD	*Fld*	Number of days from planting to 1st flowering
	Pod	Days to maturity of 1st pod (day)	PDDM	*Pddm*	Number of days from 1st flowering to harvesting of 1st pod
Yield potential	Seed	Number of seeds per pod (seeds/pod)	SDNPPD	*Sdnppd*	Number of seeds per pod

### Heritability Estimation and Correlation Analysis

The broad-sense heritability (*H*^2^) (Allard, 1999) of each trait was calculated using the following formula:

σF⁢22(σTVnu⁢ 2402+σTVNu⁢16232)/2

where $\sigma_{\mathrm{F2}}^{2},\sigma_{\text{TVNu}\,240}^{2}$ and $\sigma_{\text{TVNu}\,1623}^{2}$ are the variances of the F_2_ population, TVNu 240, and TVNu 1623, respectively. The correlation between traits was calculated with the R program (version 10.12.0).

### Quantitative Trait Loci Mapping

The F_2_ population was previously used for constructing a single nucleotide polymorphism (SNP)-based linkage map and for mapping QTLs for bruchid resistance (Amkul et al., 2019). The SNP markers were generated via a specific locus amplified fragment sequencing method (Sun et al., 2013). The map consisted of 11 linkage groups (LGs) and comprised 6,529 SNP markers (Amkul et al., 2019). The linkage map and genotypic (SNP marker) data were used for analyzing the QTLs of domestication-related traits in the present study.

The QTLs were analyzed according to the inclusive composite interval mapping (ICIM) method (Li et al., 2007) of the QTL IciMapping 4.2 software^1^, with 0.1 cM steps. A probability of stepwise regression of 0.001 was used for the ICIM. A logarithm of odds of 3.0 was used as the threshold for assessing the significance of the QTLs.

### Identification of Candidate Genes for Domestication-Related Traits

Candidate genes at the major QTLs [i.e., phenotypic variance explained (PVE) ≥10%] for domestication-related traits were identified based on the nearest DNA markers flanking the QTLs on the cowpea reference genome sequence (Lonardi et al., 2019)^2^. All of the annotated genes between the markers were identified, after which the genes with functions potentially associated with the specific trait were selected as candidate genes.

## Results

### Variation and Heritability of Domestication-Related Traits

**FIGURE 1:**
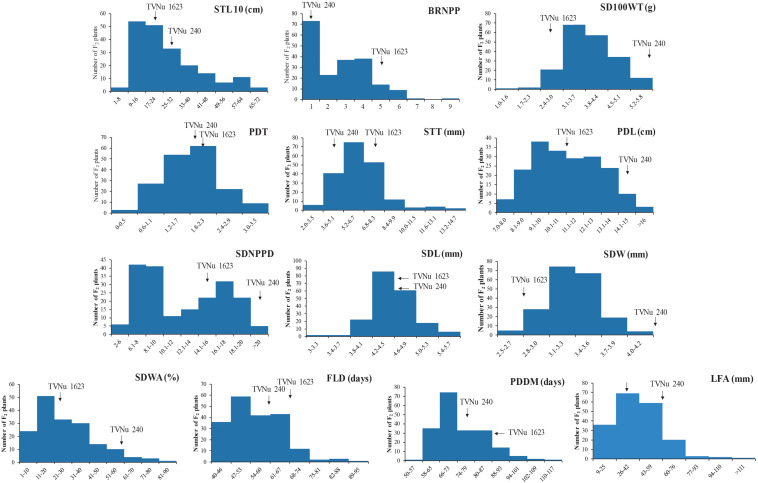
Frequency distribution of 13 domestication-related traits in the zombi pea F_2_ population developed from a cross between cultivated zombi pea (TVNu 240; *V. vexillata* var. *macrosperma*) and wild zombi pea (TVNu 1623; *V. vexillata* var. *vexillata*).

The data for the domestication-related traits of the F_2_ population (mean and range) and the traits of the parents (mean) are summarized in Table 2. The values for the following traits were higher for the cultivated parent (TVNu 240) than for the wild parent (TVNu 1623): germination (SDWA), organ size (SD100WT, LFA, SDL, PDL, and STL), yield (SDNPPD), and earliness (FLD and PDDM). In contrast, the STT and branching (BRNPP) values were higher for the wild parent than for the cultivated parent. The parents had very similar PDT and SDL values. The SDWA, SD100WT, LFA, SDW, STT, BRNPP, and SDNPPD mean values of the F_2_ population were between those of the parents (Table 2). However, the PDT and SDL mean values were similar among the F_2_ population and both parents. The F_2_ population mean values for PDL and STL were almost the same as those of the wild parent and cultivated parent, respectively, whereas the SDNPPD mean value was lower for the F_2_ population than for either parent (Table 2). All traits exhibited a continuous distribution (Figure 1), indicative of a quantitative trait inheritance. Specifically, SDNPPD exhibited a bimodal distribution, suggesting that this trait may be controlled by a few loci. Transgressive segregation was clearly detected for several traits, including PDT, SDWA, LFA, PDL, STT, STL, BRNPP, FLD, PDDM, and SDNPPD (Figure 1 and Table 2), implying that both parents possessed alleles that positively and negatively affected these traits. Most of the traits exhibited moderate to relatively high heritability (Table 2), with the heritability value exceeding 0.70 for seven traits.

**TABLE 2:** Mean, minimum, and maximum values as well as the broad-sense heritability (*H*^2^) of domestication-related traits of the zombi pea F_2_ population derived from a cross between TVNu 240 (cultivated) and TVNu 1623 (wild).

**Trait^a^**	**Unit**	**TVNu 240**	**TVNu 1623**	**F_2_ population**		***H*^2^**
			
		**Mean**	**Mean**	**Mean**	**Min–Max**	
PDT	count	1.85	1.99	1.81	0–3.3	0.72
SDWA	%	59.80	23.20	27.25	2.00–89.47	0.68
SD100WT	g	5.89	2.61	3.92	1.35–5.80	0.78
SDL	mm	4.59	4.51	4.54	3.21–5.60	0.40
SDW	mm	4.35	2.83	3.37	2.60–4.19	0.54
PDL	cm	14.55	11.13	11.24	7.12–25.86	0.92
STT	mm	4.76	7.78	6.45	2.26–13.84	0.52
STL	cm	27.50	20.25	27.12	5.50–72.00	0.83
BRNPP	count	0.00	4.20	1.66	0–8	0.42
FLD	day	59.40	67.40	55.93	40–91	0.75
PDDM	day	77.30	87.60	74.73	52–110	0.82
SDNPPD	count	20.54	15.15	12.38	2.75–21.8	0.81
LFA	mm^2^	59.65	35.35	41.80	9.44–132.98	0.64

*^*a*^Trait abbreviations are shown in Table 1.*

The correlations among the examined traits are listed in Supplementary Table S2. There were significant and positive correlations between related traits, including SD100WT and SDW or SDL, PDL and SDNPPD, and FLD and PDDM. Additionally, SD100WT was positively correlated with PDT, STT, STL, BRNPP, and LFA, but negatively correlated with SDNPPD. Interestingly, LFA was significantly and positively correlated with all of the other traits, with the exception of SDWA and PDT. Moreover, SDWA was significantly and negatively correlated with PDT, FLD, PDDM, and BRNPP. The data also revealed a significant correlation between BRNPP and all other analyzed traits.

### QTLs and Candidate Genes for Domestication-Related Traits

**FIGURE 2:**
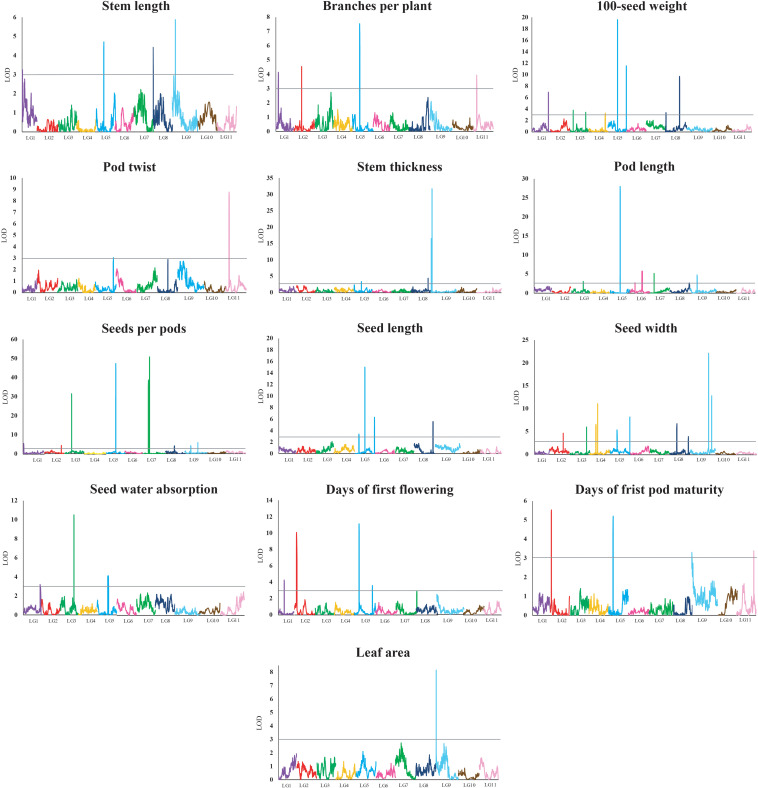
Quantitative trait locus plots for 13 domestication-related traits in the zombi pea F_2_ population developed from a cross between cultivated zombi pea (TVNu 240; *V. vexillata* var. *macrosperma*) and wild zombi pea (TVNu 1623; *V. vexillata* var. *vexillata*). The *x*-axis indicates the linkage groups, whereas the *y*-axis indicates the logarithm of odds (LOD) scores. The gray line horizontal to the *y*-axis indicates the LOD significance threshold.

A total of 62 QTLs were identified for the 13 analyzed traits, with 1–11 loci per trait (Table 3 and Figure 2). These 62 QTLs were distributed in all LGs, except for LG10. The number, location, and effect of the QTLs identified for each trait are summarized in Table 3.

**TABLE 3:** Quantitative trait loci for 13 domestication-related traits in the zombi pea F_2_ population derived from a cross between TVNu 240 (female cultivated) and TVNu 1623 (male wild).

**Trait^1^**	**QTL name**	**LG**	**Position^2^**	**LOD**	**PVE^3^ (%)**	**Add^4^**	**Dom^5^**
PDT	*qPdt5.1–*	5	137.4	3.1	5.68	–0.22	0.00
	*qPdt11.1–*	11	22.3	8.8	17.57	–0.07	–0.50
SDWA	*qSdwa1.1+*	1	142.3	3.2	6.34	1.98	7.97
	*qSdwa3.1–*	3	145.0	10.5	22.22	–10.66	–5.82
	*qSdwa5.1+*	5	88.3	4.1	8.71	6.84	–1.33
SD100WT	*qSd100wt1.1+*	1	135.1	6.9	5.46	0.25	0.06
	*qSd100wt3.1+*	3	29.3	3.8	2.89	0.19	0.01
	*qSd100wt3.1+*	3	164.0	3.4	2.57	0.16	0.08
	*qSd100wt4.1–*	4	146.2	3.6	2.76	–0.02	0.26
	*qSd100wt5.1+*	5	78.6	19.6	17.92	0.05	0.65
	*qSd100wt5.2+*	5	150.1	11.6	9.54	0.32	0.10
	*qSd100wt8.1+*	8	7.7	3.4	2.56	0.19	–0.02
	*qSd100wt8.2+*	8	112.6	9.7	7.84	0.30	0.09
LFA	*qLfa9.1–*	9	1.1	8.1	18.24	–8.69	6.75
SDL	*qSdl5.1–*	5	30.9	3.5	4.79	–0.11	–0.01
	*qSdl5.2+*	5	78.6	15.2	23.84	0.03	0.36
	*qSdl5.3+*	5	155.2	6.4	9.07	0.17	–0.04
	*qSdl8.1+*	8	154.2	5.6	7.87	0.14	0.04
SDW	*qSdw2.1+*	2	109.2	4.9	4.00	0.03	0.10
	*qSdw3.1+*	3	163.0	6.0	5.11	0.09	0.02
	*qSdw4.1+*	4	51.3	6.6	5.67	0.01	–0.15
	*qSdw4.2+*	4	65.8	11.1	10.10	0.02	0.18
	*qSdw5.1+*	5	56.3	5.4	4.51	0.03	0.12
	*qSdw5.2+*	5	156.5	8.2	7.18	0.11	0.01
	*qSdw8.1+*	8	58.1	6.8	5.81	0.10	–0.02
	*qSdw8.2+*	8	154.2	4.0	3.29	0.07	0.02
	*qSdw9.1+*	9	135.3	20.6	20.56	0.19	0.05
	*qSdw9.2–*	9	157.8	12.0	10.82	–0.14	0.03
PDL	*qPdl3.1+*	3	121.1	3.3	3.30	0.26	0.71
	*qPdl5.1–*	5	80.4	28.0	37.72	–0.03	–2.75
	*qPdl6.1–*	6	83.1	6.0	6.05	–0.68	–0.46
	*qPdl7.1+*	7	39.3	5.2	5.37	0.74	–0.16
	*qPdl9.1–*	9	48.3	4.5	4.57	–0.63	0.22
STT	*qStt5.1–*	5	64.5	3.4	5.52	–0.58	–0.10
	*qStt8.1–*	8	144.6	4.5	7.43	–0.28	–0.98
	*qStt9.1+*	9	2.9	16.2	30.63	1.48	0.24
	*qStt9.2–*	9	8.3	31.1	72.41	–2.25	–0.11
STL	*qStl1.1+*	1	4.5	3.2	5.78	1.92	6.21
	*qStl5.1–*	5	62.9	4.9	9.01	–5.82	0.17
	*qStl8.1–*	8	1.5	4.4	7.97	–2.24	–7.78
	*qStl9.1–*	9	24.2	5.6	10.13	–5.83	4.27
BRNPP	*qBrnpp1.1–*	1	23.5	4.1	5.99	–0.52	0.27
	*qBrnpp2.1–*	2	62.3	4.8	7.18	–0.58	–0.05
	*qBrnpp5.1+*	5	56.2	7.5	11.51	0.04	–1.17
	*qBrnpp11.1–*	11	20.7	3.9	5.75	–0.56	0.17
FLD	*qFld1.1–*	1	56.0	4.3	6.62	–3.59	–0.55
	*qFld2.1–*	2	16.4	10.1	16.47	–4.98	–2.48
	*qFld5.1–*	5	35.1	11.1	18.61	–5.34	2.02
	*qFld5.2+*	5	127.7	3.6	5.54	3.39	0.41
PDDM	*qPddm2.1–*	2	12.0	5.5	10.02	–4.10	–1.97
	*qPddm5.1–*	5	38.7	5.2	9.47	–3.84	0.36
	*qPddm9.1–*	9	3.0	3.2	5.24	–2.28	2.83
	*qPddm11.1–*	11	111.6	3.2	5.56	–2.96	–2.10
SDNPPD	*qSdnppd1.1–*	1	2.8	5.5	1.90	–0.80	0.68
	*qSdnppd2.1–*	2	137.2	4.4	1.83	–0.83	0.18
	*qSdnppd3.1–*	3	71.4	31.6	15.00	–0.40	–3.57
	*qSdnppd5.1–*	5	80.4	47.3	27.76	–0.40	–4.88
	*qSdnppd7.1–*	7	43.3	38.8	21.05	–3.20	–0.18
	*qSdnppd7.2+*	7	51.5	50.9	33.02	3.92	–0.13
	*qSdnppd8.1–*	8	77.3	4.3	1.50	–0.71	0.54
	*qSdnppd9.1–*	9	48.2	4.0	1.39	–0.72	0.30
	*qSdnppd9.2+*	*9*	106.8	5.5	1.99	0.08	1.33

*^1^See Table 1 for trait description. ^2^Position (centimorgan) on the linkage map. ^3^Phenotypic variance explained by the QTL. ^4^Additive effect. ^5^Dominant effect.*

The wild and cultivated parents differed considerably regarding SDWA (23.20 and 59.80%, respectively). The three QTLs detected for this trait, *qSdwa1.1+*, *qSdwa3.1-*, and *qSdwa5.1+*, were in LGs 1, 3, and 5, respectively (Table 3 and Figure 2). The QTL in LG3, *qSdwa3.1-*, had the largest effect (PVE of 22.2 %). Interestingly, at this QTL, the cultivated zombi pea alleles decreased the seed coat permeability (decreased germination). The *qSdwa1.1+* and *qSdwa5.1+* QTLs both accounted for about 9% of the total variation. At these two QTLs, the cultivated zombi pea alleles increased the seed coat permeability. The *qSdwa3.1-* region consisted of approximately 179.0 kb on cowpea chromosome 3 and included 16 genes (Supplementary Table S3). Of these genes, *Vigun03g339300*, encoding a calmodulin-binding protein-like protein, was identified as a candidate gene for this QTL. The *qSdwa5.1+* region spanned about 1.74 Mb on cowpea chromosome 5 and contained 38 genes (Supplementary Table S3), with *Vigun05g170600* and *Vigun05g170700*, both of which encode gibberellin 2-beta-dioxygenase 1, identified as candidate genes for this QTL.

The wild zombi pea exhibited extensive pod dehiscence, in contrast to the cultivated zombi pea (Table 2). Two QTLs were identified for this trait, with one each in LGs 5 and 11 (Table 3 and Figure 2). The QTL in LG11, *qPdt11.1−*, had a PVE of 17.6%, whereas the QTL in LG5, *qPdt5.1−*, had a PVE of only 5.7%. At both QTLs, the cultivated zombi pea alleles decreased pod dehiscence. The *qPdt11.1−* and *qPdt5.1−* regions spanned approximately 406 and 72.7 kb on cowpea chromosomes 11 and 5, and included eight and seven genes, respectively (Supplementary Table S3). These genes included *Vigun11g053700* [encoding the bifunctional UDP-glucose 4-epimerase and UDP-xylose 4-epimerase 1 (UGE1)] and *Vigun11g054200* [encoding glycosyltransferase family 14 (GT14)], which were considered as candidate genes for *qPdt11.1−*. The *Vigun05g241400* gene, encoding UDP-apiose/xylose synthase, was selected as a candidate gene for *qPdt5.1−*.

The cultivated zombi pea seeds were about 2.5-fold larger than the wild zombi pea seeds (Table 2). Regarding the seed size-related traits (SD100WT, SDL, and SDW), 4–10 QTLs were identified, with at least one QTL in all LGs, with the exception of LGs 10 and 11 (Table 3 and Figure 2). At all QTLs, except for *qSd100tw4.1*− and *qSdw9.1−*, the cultivated zombi pea alleles were associated with increases in seed size. The QTLs with the largest effects on SD100WT and SDL were *qSd100wt5.1+* (PVE of 17.9%) and *qSdl5.2+* (PVE of 23.8%), respectively, both of which belonged to LG5 (Table 3). The QTL that contributed the most to SDW was *qSdw9.1+* (PVE of 20.6%), which was in LG9. Additionally, LG5 and LG8 included 11 and 5 QTLs for seed size, respectively. Interestingly, *qSd100wt5.1+* and *qSdl5.2+* were mapped to the same position. Another large-effect QTL for SD100WT was *qSd100tw5.2* + (PVE of about 10%). The *qSd100wt5.1+* and *qSdl5.2+* region covered 1.03 Mb on cowpea chromosome 5 and contained 38 genes (Supplementary Table S3). The identified candidate genes included *Vigun05g139100*, which encodes an auxin-responsive protein-related protein, and *Vigun05g140300*, which encodes a protein with an unknown function (DUF538). The *qSd100wt5.2+* region comprised 217.9 kb on cowpea chromosome 5 and contained 30 genes (Supplementary Table S3). Among these genes, *Vigun05g277600*, encoding an IAA-amino acid hydrolase ILR1-like 1-related protein, was considered as a candidate gene for *qSd100wt5.2+*. Moreover, the *qSdw9.1+* region covered a 130.0-kb region on cowpea chromosome 9 and comprised 12 genes (Supplementary Table S3). Both *Vigun09g215300* (WRKY transcription factor 40-related protein) and *Vigun09g215400* (WRKY DNA-binding protein) were selected as candidate genes for this QTL.

The leaves of the cultivated parent were larger than those of the wild parent. The only QTL detected for LFA, *qLfaA9.1−* (PVE of 18.2%), was mapped to LG9 (Table 3 and Figure 2). Moreover, the cultivated parent alleles at this QTL decreased the LFA value. The *qLfa9.1−* region spanned 201.1kb on cowpea chromosome 9 and comprised 17 genes (Supplementary Table S3). Among these genes, *Vigun09g018300* and *Vigun09g017000*, encoding a WD40 repeat-containing protein and falz-related bromodomain-containing protein/transcription factor GTE1, respectively, were identified as candidate genes for this QTL.

The cultivated zombi pea pods were approximately 3.5 cm longer than the wild zombi pea pods (Table 2). Five QTLs related to PDL were detected on different LGs (Table 3 and Figure 2). At QTLs *qPdl3.1+* and *qPdl7.1+*, the cultivated zombi pea alleles increased the PDL value. Additionally, at QTLs *qPdl5.1*−, *qPdl6.1*−, and *qPdl9.1*−, the wild zombi pea alleles increased the PDL value. The *qPdl5.1−* QTL in LG5 had a PVE of 37.7%, which was considerably higher than the PVE for the other four QTLs (<5%). The *qPdl5.1−* region spanned 860.4 kb on cowpea chromosome 5 and contained 41 genes (Supplementary Table S3). The *Vigun05g125800* gene, encoding pectate lyase 1, was chosen as a candidate gene for *qPdl5.1−*.

The cultivated zombi pea plants had shorter stems (the first 10 internodes), but more branches, than the wild zombi pea plants. The four QTLs for STL belonged to different LGs (Table 3 and Figure 2). The PVE of each of the QTLs for STL was ≤10%, but was highest for *qStl9.1*− in LG9. The cultivated parent alleles at three of the four QTLs decreased the internode length. Similarly, four QTLs on different LGs were detected for BRNPP, and the PVE of each QTL was ≤12%, but was highest for *qBrnpp5.1*− in LG5 (Table 3 and Figure 2). The cultivated parent alleles at three of the four QTLs decreased the number of branches. Only one marker flanking *qStl9.1*−, Marker293030, matched a sequence in the cowpea reference genome. The *Vigun09g037900* gene, encoding a MOB-like protein phocein (MOB1), which was detected approximately 113 kb from Marker293030, was considered to be a candidate gene for *qStl9.1*−. The *qBrnpp5.1*− region was less than 1.0 kb on cowpea chromosome 5 and included *Vigun05g082200*, which encodes an auxin efflux carrier family protein (Supplementary Table S3).

Domestication generally leads to cultivated crops that flower and mature earlier than their wild progenitors. The cultivated zombi pea plants flowered and matured approximately 10 and 8 days earlier, respectively, than the wild zombi pea plants (Table 2). Four QTLs on three LGs were identified for FLD (Table 3 and Figure 2). The effects of QTLs *qFld2.1−* in LG2 and *qFld5.1−* in LG5 on FLD were similar (PVE of 16.5 and 18.6%, respectively). With the exception of *qFld5.2+*, the cultivated parent alleles at these QTLs enhanced flowering. The *qFld2.1−* region spanned 391.5 kb on cowpea chromosome 2 and comprised 28 genes (Supplementary Table S3). Both *Vigun02g051100* (WRKY transcription factor 57) and *Vigun02g052800* (ring finger and CCH-type zinc finger domain-containing protein) were selected as candidate genes for *qFld2.1−*. An analysis of *qFld5.1−* revealed that this QTL spanned 90.5 kb on cowpea chromosome 5 and contained eight genes (Supplementary Table S3), of which *Vigun05g043100*, encoding a bHLH transcription factor PRE1-related protein, was identified as a candidate gene.

Similarly to the FLD analysis, four QTLs on different LGs were identified for PDDM (Table 3 and Figure 2). The *qPddm2.1−* (LG2) and *qPddm5.1−* (LG5) QTLs had the largest effects on this trait (PVE of about 10%). At all four QTLs, the cultivated parent alleles accelerated maturity. Additionally, *qFld2.1−* and *qPddm2.1−* were located close to each other, whereas *qFld5.1−* was mapped close to *qPddm5.1−*. The *qPddm2.1−* region covered 83.3 kb on cowpea chromosome 2 and contained seven genes, including *Vigun02g050200*, encoding the 14-3-3-like protein GF14 IOTA, which was chosen as a candidate gene. The *qPddm5.1−* region spanned only 19.0 kb on cowpea chromosome 5 and contained four genes (Supplementary Table S3). The *Vigun05g050300* gene, which encodes 3-deoxy-8-phosphooctulonate synthase/phospho-2-keto-3-deoxyoctonate aldolase, was designated as a candidate gene for *qPddm5.1−*.

The cultivated parent produced more seeds per pod than the wild parent. Nine QTLs were detected for SDNPPD (Table 3 and Figure 2). The *qSdnppd3.1−*, *qSdnppd5.1−*, *qSdnppd7.1−*, and *qSdnppd7.2+* QTLs had larger effects on SDNPPD (PVE of 15.0, 27.8, 21.1, and 33.0%, respectively) than the other QTLs (PVE of only about 2.0%). Regarding the *qSdnppd7.2+* and *qSdnppd9.2+* QTLs, the cultivated zombi pea alleles increased the seed yield. In contrast, at the other QTLs, the cultivated zombi pea alleles had the opposite effect on seed yield. The *qSdnppd3.1−* region covered about 221.6 kb on cowpea chromosome 3 and comprised five genes, including *Vigun03g187300*, which encodes abscisic acid (ABA)-insensitive 5-like protein 6. The *qSdnppd5.1−* and *qSdnppd7.2+* regions spanned approximately 860.4 kb and 2.38 Mb on cowpea chromosomes 5 and 7 and contained 41 and 60 genes, respectively. Moreover, *Vigun05g126900*, encoding the MALE STERILE 5 protein, was identified as a candidate gene for *qSdnppd5.1−* (Supplementary Table S3). Both *Vigun07g094201* and *Vigun07g096300*, encoding the B3-like transcription factor and the B3 DNA-binding domain protein, respectively, were selected as candidate genes for *qSdnppd7.2+* (Supplementary Table S3). An analysis of *qSdnppd7.1−*, revealed only one flanking marker (Marker225965) on cowpea chromosome 7. Furthermore, *Vigun07g074900*, encoding the transcription factor TCP21, and located approximately 81.6 kb from Marker225965, was identified as a candidate gene for *qSdnppd7.1−*.

## Discussion

Compared with wild zombi pea, cultivated zombi pea has undergone phenotypic changes due to domestication. In this study, we identified 62 QTLs controlling 13 domestication-related traits in zombi pea. The QTLs were distributed in all LGs, except for LG10. The number of QTLs identified for individual traits varied from one to ten, with an average of five QTLs (Table 3). Large-effect QTLs (PVE > 15%) were detected for all examined traits, except for STL, PDDM, and BRNPP (Table 3). These results imply that the zombi pea domestication-related traits are controlled by one or only a few major QTLs along with a small number of minor QTLs. This is consistent with the results of earlier investigations of other *Vigna* species, including mung bean (Isemura et al., 2012), azuki bean (Isemura et al., 2007), rice bean (Isemura et al., 2010), and cowpea (Kongjaimun et al., 2012; Lo et al., 2018). However, in a recent study, Dachapak et al. (2018) identified an average of 2.3 QTLs for zombi pea domestication-related traits, which is about twofold less than the number of QTLs detected in our study. This difference may have been due to the sterility of the mapping population used in the earlier study.

In *Vigna* species, the major QTLs for domestication-related traits, especially organ size, have always been mapped to the same LG (Isemura et al., 2007, 2010, 2012; Kongjaimun et al., 2012; Lo et al., 2018; Yundaeng et al., 2019). In our study, the major QTLs for the domestication-related traits of zombi pea, including SD100WT, SDL, PDL, STL, BRNPP, FLD, PDDM, and SDNPPD, were clustered in LG5, corresponding to cowpea chromosome 5. In an earlier study of cowpea, which is the legume crop most closely related to zombi pea, the major QTLs for domestication-related traits, such as pod shattering, seed dormancy, seed size (weight, width, and length), pod size (length and width), stem size (thickness and length), and leaf size (width and length), were clustered in LG7 (Kongjaimun et al., 2012), which corresponds to chromosome 5. These findings suggest that the genetics underlying the effects of domestication on the organ size (e.g., seed size, pod length, and stem length) of these two African *Vigna* crops may be the same. However, the genetic basis of seed dormancy and pod shattering, which are key domestication-related traits, varies between these two species.

Domestication results in a decrease in or a loss of seed dormancy, enabling the uniform germination of seeds, which is relevant for agricultural production. The seed dormancy of cultivated zombi pea is suppressed. In this study, we identified one major and two minor QTLs for the seed dormancy-related trait (Table 3). Specifically, *qSdwa3.1−* was detected as the major QTL, accounting for 22.2% of the phenotypic variation. Because the wild alleles at *qSdwa3.1−* positively affected SDWA (i.e., decreased dormancy), their introgression into cultivated zombi pea may be useful for decreasing seed dormancy. The *Vigun03g339300* gene, encoding a calmodulin-binding protein-like protein, was identified at *qSdwa3.1−*. Calmodulin (CaM) and CaM-like (CML) proteins are the major Ca^2+^-binding proteins. The CaM/CML signals are involved in the ABA-induced inhibition of seed germination and seedling growth (Zhou et al., 2018). In *Arabidopsis thaliana* (Arabidopsis), IQM4, which is a CaM-binding protein, was recently reported to affect seed dormancy by modulating ABA biosynthesis and ABA signaling during seed maturation and germination (Zhou et al., 2018). In cowpea, *Sdp1.1+* is the major QTL related to seed dormancy, explaining about 40% of the trait variation (Kongjaimun et al., 2012). The markers flanking *Sdp1.1+* suggest that this QTL is located on chromosome 3 between positions 45,527,961 and 51,968,469. Thus, *qSdwa3.1−* and *Sdp1.1+* are different loci. The minor QTL *qSdwa5.1+* accounted for about 9% of the phenotypic variation, and included *Vigun05g170600* and *Vigun05g170700*, both of which encode gibberellin 2-beta-dioxygenase 1 (GA2OX1). Gibberellin is a key plant hormone that regulates seed dormancy and germination by antagonistically suppressing ABA-triggered seed dormancy (Shu et al., 2016). Loss-of-function mutations to *GA2ox2*, *GA2ox7*, and *GA2ox8*, which encode GA2OX, reportedly enhance seed germination in Arabidopsis (Schomburg et al., 2003; Yamauchi et al., 2007).

Preventing or limiting pod dehiscence (pod shattering) may minimize pre-harvest yield losses, resulting in a more efficient harvest. Thus, pod indehiscence may be an advantageous trait during the harvesting of seeds, making it an important consideration during crop domestication. A major QTL in LG11 and a minor QTL in LG5 were related to pod twisting (Table 3). The major QTL, *qPdt11.1+*, explained 17.6% of the dehiscence variation, and included two genes, *Vigun11g053700*, encoding UGE1, and *Vigun11g054200*, encoding the glycosyltransferase GT14. Earlier studies confirmed that UGE is a nucleotide sugar interconversion enzyme that mediates the interconversion of UDP-D-glucose and UDP-D-galactose and contributes to cell wall biosynthesis (Barber et al., 2006; Rösti et al., 2007). Glycosyltransferases in plants are mainly involved in the biosynthesis of cell wall polysaccharides and glycoproteins (Hansen et al., 2012). Although the functions of plant GT14 remain uncharacterized, two *GT14* genes identified in a hybrid aspen species (*Populus tremula* × *P. tremuloides*) reportedly exhibit xylem-specific expression (Aspeborg et al., 2005). Accordingly, GT14 may participate in secondary cell wall biosynthesis. In cowpea, three major QTLs for pod shattering have been identified (Kongjaimun et al., 2012; Suanum et al., 2016; Lo et al., 2018). Candidate genes for these QTLs include *Vigun02g095200* (cellulose synthase), *Vigun03g306000* (NAC domain transcription factor), *Vigun03g302600* (C2H2 zinc finger protein), *Vigun05g262100* (MYB46 transcription factor), *Vigun05g273500* (MYB26 transcription factor), and *Vigun05g266600* (beta glucosidase) (Suanum et al., 2016; Lo et al., 2018; Takahashi et al., 2019; Watcharatpong et al., 2020). Thus, the major QTLs/genes which control pod shattering differ between zombi pea and cowpea.

In most cases, domestication increases organ sizes, especially the harvested or consumed or exploited organs. Seed size is a key determinant of seed yield in legume crops as well as evolutionary fitness. It is a polygenic trait affected by various cellular processes. Twenty-two QTLs were identified for seed size-related traits, of which the QTLs with the largest effects on seed weight (*qSd100wt5.1+*) and seed length (*qSdl5.1+*) were mapped to the same position of LG5 (Table 3). Two genes were identified at these two QTLs, namely *Vigun05g139100*, encoding an auxin-responsive protein-related protein, and *Vigun05g140300*, encoding DUF538. Auxin is important for seed development, with specific effects on the embryo, endosperm, and seed coat, all of which influence seed size and yield (Cao et al., 2020). Genes encoding auxin-related proteins control seed size (Schruff et al., 2006; Ishimaru et al., 2013; Liu et al., 2015; Sun et al., 2017). In Arabidopsis mutant line F08314, the overexpression of *RAFL06-12-J05* (encoding DUF538) results in seeds that are larger than the wild-type seeds (Akiyama et al., 2014)^3^. In addition to *qSd100wt5.1+*, *qSd100wt5.2+* also substantially affects seed weight. We identified *Vigun05g277600*, which encodes an IAA-amino acid hydrolase ILR1-like 1-related protein, as a candidate gene for *qSd100wt5.2+*. Earlier studies revealed ILR1 is involved in auxin conjugation and affects auxin homeostasis (Rampey et al., 2004; and see Cao et al., 2020 for review). In rice, *TGW3*, encoding an IAA-glucose hydrolase, controls the rice grain weight and yield (Ishimaru et al., 2013). The QTL with the largest effect on seed length, *qSdl9.1+*, was included in LG9. Both *Vigun09g215300* (WRKY transcription factor 40-related protein) and *Vigun09g215400* (WRKY DNA-binding protein) were detected as candidate genes for *qSdl9.1+*. In plants, WRKY proteins have diverse biological functions related to seed development as well as developmental and hormone-controlled processes (Bakshi and Oelmüller, 2014). For example, *MINISEED3*, which encodes WRKY10, regulates seed size in Arabidopsis (Luo et al., 2005) and *Loose Panicle1*, which encodes a WRKY transcription factor, regulates seed size by increasing the seed length and width in foxtail millet [*Setaria italica* (L.) P. Beauv.] (Xiang et al., 2017). Additionally, *SoyWRKY15a*, which encodes WRKY15, is associated with changes to seed size due to the domestication of soybean [*Glycine max* (L.) Merr.], with expression levels that vary significantly between domesticated and wild soybeans (Gu et al., 2017).

In the current study, *qLfa9.1−*, with a PVE of 18.2%, was the only QTL identified for leaf size (Table 3). The *Vigun09g018300* gene (WD40 repeat-containing protein) was detected as a candidate gene for this QTL. In cucumber, *LITTLELEAF*, which encodes a WD40 repeat domain−containing protein, regulates the size of various organs, including leaves (Yang et al., 2018). We also identified *Vigun09g017000*, encoding the bromodomain-containing protein GTE1, as a candidate gene. In Arabidopsis, *GTE4* and *GTE6* encode bromodomain-containing proteins that regulate leaf shape (and thus leaf size). Specifically, GTE4 controls the mitotic cell cycle during plant development (Airoldi et al., 2010), whereas GTE6 induces the expression of *ASYMMETRIC LEAVES1*, encoding a MYB-domain protein that regulates proximodistal leaf patterns (Chua et al., 2005).

Legume domestication results in increased pod length. Five QTLs were identified for pod length, but *qPdl5.1−* was the only major QTL, accounting for nearly 38% of the trait variation (Table 3). Pod elongation requires changes to fiber cell structures and components. The *Vigun05g125800* gene, encoding pectate lyase 1, was selected as a candidate gene for *qPdl5.1−*. Pectin is a major polymer in the plant primary cell wall. Pectate lyase is a pectin-degrading enzyme that can degrade demethylesterified homogalacturonan (HG), which is a pectic polymer (Palusa et al., 2007). The methylesterification status of HG can regulate cellular growth and cell shape, thereby influencing plant growth and development (Wolf et al., 2009; Peaucelle et al., 2011). A previous study regarding common bean (*Phaseolus vulgaris* L.), which is a legume closely related to zombi pea, revealed that the major changes in cell wall polysaccharides during pod development involve pectic polymers and that the deposition of galactose-rich pectic polymers in cell walls increases in elongating pods (Stolle-Smits et al., 1999). Moreover, during pod elongation, the HG level steadily increases. Accordingly, pectate lyase may help mediate the elongation of zombi pea pods. In rice, a mutation to *DEL1*, which encodes a pectate lyase precursor, decreases the total pectate lyase activity, leading to an increase in the methylesterified HG content and altered cell wall compositions and structures, with implications for plant height, root length, flag leaf length, grain size, and panicle and internode lengths. In cotton, the downregulated expression of pectate lyase genes *GhPEL48_Dt* and *GhPEL76* decreases fiber length by impeding pectin degradation (Wang et al., 2009; Sun et al., 2020).

Most domesticated legume crops exhibit greater determinate growth, with less branching, than their wild ancestors. The *qStl9.1*− QTL explained about 10% of the variability in the stem length (plant height) (Table 3). We identified *Vigun09g037900*, encoding MOB1, as a candidate gene for this QTL. Arabidopsis MOB1 proteins are critical for plant development because they promote auxin signaling (Cui et al., 2016) and regulate jasmonate accumulation (Guo et al., 2020). Mutations to the *MOB1* gene in Arabidopsis negatively affect plant height (Xiong et al., 2016; Guo et al., 2020). We identified *Vigun05g082200*, encoding an auxin efflux carrier protein, as a candidate gene for *qBrnpp5.1*−, which controls the number of branches in zombi pea. The Vigun05g082200 sequence is similar to that of the PIN-LIKES 6 transporter (AT5G01990). The PIN proteins form a family of auxin efflux carriers. Auxin is well known for its ability to regulate numerous plant growth and developmental processes such as meristem formation and lateral organ formation and patterning (Wang and Jiao, 2018). For example, PIN6 contributes to auxin signaling-mediated developmental processes, including lateral/adventitious root organogenesis, primary/lateral root development and growth, and shoot apical dominance (Cazzonelli et al., 2013; Nisar et al., 2014; Simon et al., 2016). In rice, *PIN1* and *PIN2* are involved in tillering (Xu et al., 2005; Chen et al., 2012).

Generally, wild ancestors of domesticated crops are sensitive to short-day conditions. In this study, we identified four QTLs related to days-to-flowering in zombi pea, of which two are large-effect QTLs, *qFld2.1−* and *qFld5.1−* (PVE of 16.5 and 18.6%, respectively). We selected *Vigun02g051100* (WRKY57 transcription factor) and *Vigun02g052800* (RING finger and CCCH-type zinc finger protein) as candidate genes for *qFLD2.1−* and *qFLD5.1−*, respectively. The WRKY transcription factors are involved in physiological changes and responses to biotic and abiotic stresses. In Arabidopsis, WRKY12, WRKY13, WRKY71, and WRKY75 regulate flowering time (Li W. et al., 2016; Yu et al., 2016; Zhang et al., 2018). Additionally, WRKY75 interacts with DELLA proteins, which are the major components involved in gibberellic acid perception and signaling (Zhang et al., 2018). A mutation to *WRKY75* reportedly delays flowering, whereas the overexpression of this gene has the opposite effect (Zhang et al., 2018). Plant zinc finger proteins comprise a large protein family and have diverse functions related to plant development and resistance to biotic and abiotic stresses. The Arabidopsis genes *AtC3H17*, *AtZFP1*, *AtKHZ1*, and *AtKHZ2* as well as the *Medicago sativa* gene *MsZFN*, encoding CCCH zinc finger proteins, influence flowering time (Chao et al., 2014; Seok et al., 2016; Yan et al., 2017; Wang et al., 2018).

Four QTLs were detected for days-to-flowering in zombi pea, of which *qPddm2.1−* and *qPddm5.1−* had a similar PVE of about 10% (Table 3). We identified *Vigun02g050200*, encoding the 14-3-3-like protein GF14 IOTA, and *Vigun05g050300*, encoding 3-deoxy-8-phosphooctulonate synthase/phospho-2-keto-3-deoxyoctonate aldolase, as the candidate genes for *qPddm2.1−* and *qPddm5.1−*, respectively. The 14-3-3 (GF14) proteins are critical components of various cellular signaling processes and are important regulators of several physiological processes in plants. The *GF14* genes are associated with fruit development and/or ripening in banana (*Musa acuminata* L.) (Li et al., 2012; Li M. et al., 2016) and pear (*Pyrus pyrifolia* Nakai.) (Shi and Zhang, 2014). Phospho-2-keto-3-deoxyoctonate aldolase is also known as 3-deoxy-d-manno-2-octulosonic acid-8-phosphate synthase (KDO8P), which has a key function related to the biosynthesis of KDO, which is a sugar present only in the rhamnogalacturonan II pectic fraction of the primary cell walls of higher plants (Delmas et al., 2003). In the common bean, which is a legume closely related to zombi pea, pod elongation and maturation are accompanied by changes to rhamnogalacturonan (Stolle-Smits et al., 1999).

The number of seeds per pod is one of the yield determinants of legumes. Some domesticated legumes have more seeds per pod than their wild ancestors. Up to nine QTLs were detected for this trait in zombi pea (Table 3). Among these QTLs, *qSdnppd3.1−*, *qSdnppd5.1−*, *qSdnppd7.1−*, and *qSdnppd7.2+* had relatively large effects (PVE between 15 and 33%). Because the wild alleles at *qSdnppd3.1−*, *qSdnppd5.1−*, and *qSdnppd7.1−* were observed to increase the number of seeds per pod, they may be useful for increasing the seed yield of cultivated zombi pea. We identified *Vigun03g187300* (ABA-insensitive 5-like protein 6) as a candidate gene for *qSdnppd3.1−*. The encoded protein is an ABA-responsive element (ABRE)-binding factor that regulates ABRE-dependent gene expression (Nakashima and Yamaguchi-Shinozaki, 2013). In Arabidopsis, an ABA-deficiency reportedly decreases the number of seeds per silique (Cheng et al., 2014). The *Vigun05g126900* gene, encoding MALE STERILE 5, was selected as a candidate gene for *qSdnppd5.1−*. In an earlier investigation on Arabidopsis, mutations to *MS5*, which encodes MALE STERILE 5, resulted in the formation of “polyads” (i.e., tetrads with more than four pools of chromosomes following male meiosis) (Glover et al., 1998). Plants that are homozygous for the *ms5* recessive allele reportedly exhibit stunted growth and produce empty siliques, whereas in plants that are heterozygous for *MS5*, silique elongation and seed set are less inhibited (Glover et al., 1998). We identified *Vigun07g094201* (B3-like transcription factor) and *Vigun07g096300* (B3 DNA-binding domain protein) as the candidate genes for *qSdnppd7.1−*. The B3 domain family proteins are associated with ovule development (Skinner and Gasser, 2009). A recent study on Arabidopsis investigated the roles of three *Reproductive Meristem* (*REM*) genes, *REM34*, *REM35*, and *REM36*, which encode B3 domain family transcription factors affecting female and male gametophyte development (Caselli et al., 2019). This previous study proved that the silencing of one or more of these genes via RNA interference increases the number of unfertilized ovules, thereby decreasing the number of seeds per silique. Additionally, the Vigun07g094201 sequence is highly similar to that of Arabidopsis REM39 (AT3G18990). In the current study, *Vigun07g074900*, which encodes the transcription factor TCP21, was selected as a candidate gene for *qSdnppd7.2+*. In Arabidopsis, several TCP transcription factors, including TCP21, are involved in ovule development via their interactions with SPOROCYTELESS (Chen et al., 2014; Wei et al., 2015), which is required for sporogenesis in both male and female organs (Yang et al., 1999). An earlier study involving cowpea identified a gene encoding TCP5 as a candidate gene for the number of seeds per pod (Lo et al., 2018).

## Data Availability Statement

All datasets generated for this study are included in the article/Supplementary Material.

## Author Contributions

PS and LW conceptualized the study and secured research funding. PS designed and supervised the study and revised the manuscript. KA and KL developed population and conducted data collection and analysis. PS and KA wrote the manuscript. All authors read and approved the accepted manuscript.

## Conflict of Interest

The authors declare that the research was conducted in the absence of any commercial or financial relationships that could be construed as a potential conflict of interest.

## Footnotes

1 http://www.isbreeding.net

2 https://phytozome-next.jgi.doe.gov/info/Vunguiculata_v1_2

3 http://rarge-v2.psc.riken.jp/cdna/RAFL06-12-J05

## Supplementary Material

TABLE S1 Number of markers, marker length, and distance between markers in each linkage group of the zombi pea genetic map used for detecting the QTLs of domestication-related traits in the F_2_ population derived from a cross between TVNu 240 and TVNu 1623. The markers are SNPs generated by specific locus amplified fragment sequencing. The map was constructed by Amkul et al. (2019).

TABLE S2 Correlations among domestication-related traits in the zombi pea F_2_ population derived from a cross between TVNu 240 and TVNu 1623.

TABLE S3 Candidate genes for each QTL.
