# Supplementary material for: Identification of QTLs for Domestication-Related Traits in Zombi Pea [Vigna vexillata (L.) A. Rich], a Lost Crop of Africa
Source: Front Genet. 2020 Sep 18;11:803. doi: 10.3389/fgene.2020.00803 (PMC7530282; doi:10.3389/fgene.2020.00803)
Supplement: TABLE S1 — Number of markers, marker length, and distance between markers in each linkage group of the zombi pea genetic map used for detecting the QTLs of domestication-related traits in the F2 population derived from a cross between TVNu 240 and TVNu 1623. The markers are SNPs generated by specific locus amplified fragment sequencing. The map was constructed by Amkul et al. (2019). [file Data_Sheet_1.PDF]

Supplementary Table S1. Number of markers, marker length, and distance between markers in each linkage group of the zombi pea genetic map used for detecting the QTLs of domestication-related traits in the F_2 population derived from a cross between TVNu 240 and TVNu 1623. The markers are SNPs generated by specific locus amplified fragment sequencing. The map was constructed by Amkul et al. (2019).

**Linkage group**	**No. of markers**	**Length (cM)**	**Average distance between markers (cM)**
1	546	151.6	0.28
2	494	157.4	0.32
3	1,080	193.1	0.18
4	643	167.3	0.26
5	440	156.7	0.36
6	470	136.3	0.29
7	773	188.3	0.24
8	517	168.7	0.33
9	882	184.4	0.21
10	267	108.5	0.41
11	417	128.5	0.31
**Total**	**6,529**	**1,740.8**	**0.27**
