# Supplementary material for: Identification of QTLs for Domestication-Related Traits in Zombi Pea [Vigna vexillata (L.) A. Rich], a Lost Crop of Africa
Source: Front Genet. 2020 Sep 18;11:803. doi: 10.3389/fgene.2020.00803 (PMC7530282; doi:10.3389/fgene.2020.00803)
Supplement: TABLE S2 — Correlations among domestication-related traits in the zombi pea F2 population derived from a cross between TVNu 240 and TVNu 1623. [file Data_Sheet_2.PDF]

Supplementary Table S2. Correlations among domestication-related traits in the zombi pea F_2 population derived from a cross between TVNu 240 and TVNu 1623.

	**SDWP**	**PDDM**	**FLD**	**SDW**	**SDL**	**SDNPPD**	**PLD**	**SD100WT**	**PDT**	**STT**	**BRNPP**	**STL**
**PDDM**	-0.17*											
**FLD**	-0.19*	0.67***										
**SDW**	0.08	-0.12	-0.01									
**SDL**	-0.07	-0.04	0.05	0.60***								
**SDNPPD**	-0.11	0.19**	0.18**	-0.26***	-0.42***							
**PLD**	-0.15	0.24***	0.26***	-0.02	-0.11	0.77***						
**SD100WT**	0.07	-0.08	-0.03	0.76***	0.75***	-0.39***	-0.04					
**PDT**	-0.27***	0.08	0.12	-0.26**	-0.22**	0.53***	0.48***	-0.34***				
**STT**	-0.13	0.24***	0.22**	-0.20**	0.002	0.17*	0.19**	-0.13	0.26***			
**BRNPP**	-0.19*	0.27***	0.22**	-0.33***	-0.25***	0.37***	0.31***	-0.33***	0.33***	0.48***		
**STL**	-0.04	0.13	0.20**	-0.08	0.09	0.17*	0.25***	0.05	0.23**	0.34***	0.13	
**LFA**	-0.14	0.22**	0.22**	0.22**	0.28***	0.17*	0.35***	0.31***	0.12	0.34***	0.23**	0.35***

See trait abbreviation in Table 1

*, ** and *** indicate statistically significant at probability level of 0.05, 0.01 and 0.001, respectively
